# Dehydroquinate Synthase Directly Binds to Streptomycin and Regulates Susceptibility of *Mycobacterium bovis* to Streptomycin in a Non-canonical Mode

**DOI:** 10.3389/fmicb.2022.818881

**Published:** 2022-04-19

**Authors:** Wenping Wei, Junjie Qiao, Xiaofang Jiang, Luxia Cai, Xiaomin Hu, Jin He, Min Chen, Min Yang, Tao Cui

**Affiliations:** ^1^College of Life Science and Technology, Huazhong Agricultural University, Wuhan, China; ^2^Key Laboratory of Molecular Biophysics of the Ministry of Education, College of Life Science and Technology, Huazhong University of Science and Technology, Wuhan, China; ^3^Research and Development Institute of Northwestern Polytechnical University in Shenzhen, Shenzhen, China; ^4^School of Life Sciences, Northwestern Polytechnical University, Xi’an, China

**Keywords:** antimicrobial resistance, DHQS, *aroB*, streptomycin, mycobacteria

## Abstract

Antimicrobial resistance (AMR) represents one of the main challenges in Tuberculosis (TB) treatment. Investigating the genes involved in AMR and the underlying mechanisms holds promise for developing alternative treatment strategies. The results indicate that dehydroquinate synthase (DHQS) regulates the susceptibility of *Mycobacterium bovis* BCG to first-line anti-TB drug streptomycin. Perturbation of the expression of *aroB* encoding DHQS affects the susceptibility of *M. bovis* BCG to streptomycin. Purified DHQS impairs *in vitro* antibacterial activity of streptomycin, but did not hydrolyze or modify streptomycin. DHQS directly binds to streptomycin while retaining its own catalytic activity. Computationally modeled structure analysis of DHQS–streptomycin complex reveals that DHQS binds to streptomycin without disturbing native substrate binding. In addition, streptomycin treatment significantly induces the expression of DHQS, thus resulting in DHQS-mediated susceptibility. Our findings uncover the additional function of DHQS in AMR and provide an insight into a non-canonical resistance mechanism by which protein hijacks antibiotic to reduce the interaction between antibiotic and its target with normal protein function retained.

## Introduction

Tuberculosis (TB), caused by the human pathogen *Mycobacterium tuberculosis*, remains one of the top killers worldwide (Pai et al., 2016; Aldridge et al., 2021; Singh and Chibale, 2021). The emergence and spread of drug-resistant TB have been one of the main challenges for TB treatment (Zhang et al., 2013; Desjardins et al., 2016; Manson et al., 2017; Coll et al., 2018). The long-term treatment in turn raises the risks of antibiotic resistance development. To meet the challenges, it is essential to identify the genes involved in antibiotic resistance and investigate the underlying molecular mechanisms (Wright, 2007; Lewis, 2013; Blair et al., 2015).

Aminoglycoside antibiotics streptomycin, which was discovered in 1943 from *Streptomyces griseus*, is the first effective anti-TB antibiotic and remains to be one of the essential first-line anti-TB drugs. Streptomycin binds to the 16S rRNA of bacterial 30S ribosome subunit, thus inhibiting bacteria by interfering protein synthesis (Poehlsgaard and Douthwaite, 2005). Several streptomycin resistance mechanisms have been reported. For example, mutations of three genes, namely, *rrs*, *rpsL*, and *gidB* confer *M. tuberculosis* resistance to streptomycin. The *rrs*, *rpsL*, and *gidB* genes encode 16S rRNA, ribosomal protein S12, and 16S rRNA-specific methyltransferase, respectively. The mutations of these genes directly or indirectly affect the binding of streptomycin to its 30S ribosome target. Specifically, 16S rRNA and S12 protein are the two components directly interacting with streptomycin. Therefore, mutation of 16S rRNA and S12 protein directly affects the streptomycin binding activity (Coll et al., 2018). Although it is not the target directly interacting with streptomycin, GidB can specifically methylate the N7 guanine at the position 518 of 16S rRNA directly interacting with streptomycin (Okamoto et al., 2007). Therefore, mutation of *gidB* indirectly affects the interaction between streptomycin and 30S ribosome target (Wilson, 2014). Drug modification-based resistance mechanisms have also been reported in other species, including adenylylation and phosphorylation modifications of streptomycin (Morar and Wright, 2010; Stern et al., 2018; Alekseeva et al., 2019). However, the specific genetic determinants involved in regulating intrinsic resistance to streptomycin are largely unknown in *M. tuberculosis*. It is worth noting that by whole-genome sequencing of clinical isolates, many strains have been identified to have phenotypic streptomycin resistance, but exhibit no known streptomycin resistance-associated mutations (Desjardins et al., 2016), implying that other genes might be involved in streptomycin resistance.

3-dehydroquinate synthase (DHQS), encoded by *aroB* gene, is a key enzyme in the second step of the shikimate pathway to be involved in the synthesis of aromatic amino acids. DHQS is an NAD^+^- and Zn^2+^-dependent enzyme, and it catalyzes the conversion from 3-deoxy-D-arabino-heptulosonate 7-phosphate (DAHP) to dehydroquinate (DHQ; de Mendonça et al., 2007). DHQS catalyzes five individual reactions (alcohol oxidation, phosphate v-elimination, carbonyl reduction, ring opening, and intramolecular aldol condensation) at the same active site (Carpenter et al., 1998). Structure analyses reveal that DHQS comprises an N-terminal NAD^+^ binding domain (domain N) and a C-terminal domain containing most of the residues involved in catalysis, substrate, and Zn^2+^ binding (domain C; Carpenter et al., 1998; Nichols et al., 2003). DHQS is expected to be one of the targets for novel antibacterial drug designs, since the shikimate pathway is absent in mammals, but it is required for pathogenic virulence (Liu et al., 2008; Zhu et al., 2018b; Neetu et al., 2020).

We utilized the constructed previously *Mycobacterium bovis* BCG overexpression library and screened the overexpressing strains on 7H10 plates containing 0.125 μg/ml STR and identified DHQS, which is associated with STR susceptibility. In the current study, we demonstrate that DHQS regulates the intrinsic resistance of *M. bovis* BCG to streptomycin. DHQS directly binds to streptomycin, but retains catalytic activity. Thus, we speculate that DHQS can act as a streptomycin hijacker to reduce toxicity caused by streptomycin–ribosome interaction. Considering that the DHQS sequence of *M. bovis* BCG and *M. tuberculosis* is 100% identical, it could be concluded that DHQS plays the same role in human pathogen *M. tuberculosis*.

## Materials and Methods

### Strains, Enzymes, Plasmids, and Reagents

*Escherichia coli* DH5α and BL21 (DE3) cells (purchased from Novagen, Germany) were used for gene cloning and protein expression, respectively. *Mycobacterium smegmatis* MC^2^ 155 and *M. bovis* BCG strains (purchased from ATCC) were incubated at 37°C in Middlebrook 7H9 medium containing 0.05% glycerol and 0.025% Tween-80 or in 7H10 plates containing 0.05% glycerol. Restriction enzymes, T4 DNA ligase, dNTP, rTaq DNA polymerase, DNaseI, and all the relevant buffer were purchased from New England Biolabs (United States) and TaKaRa Biotech (Shiga, Japan). Isoniazid (INH, CAS 54-85-3), ethambutol (EMB, CAS 1070-11-7), rifampicin (RIF, CAS 13292-46-1), streptomycin sulfate (STR, CAS 3810-74-0), and anhydrotetracycline (ATc, CAS 13803-65-1) were purchased from Sigma (United States). All the plasmids and strains constructed in this study were listed in Supplementary Table 1. The primers used in this study were synthesized by Tsingke Biological Technology (Wuhan, China; Supplementary Table 2).

### Cloning, Expression, and Purification of Recombinant Proteins

*Mycobacterium tuberculosis*-related genes were amplified by PCR using their respective primers with *Mycobacterium* genomic DNA as a template (Supplementary Table 2). Mutants were produced by site-directed mutagenesis using Mut Express II Mutagenesis Kit (Vazyme, Nanjing, China). Then the amplified DNA fragments were cloned into the prokaryotic expression vector pET28a, and the vector was transferred into the BL21 expression strain. The recombinant strains were grown in 400 ml LB liquid medium at 37°C until OD_600_ reached 0.6. DHQS was induced at 30°C for 4–5 h with 0.4 mM isopropyl β-D-thiogalactoside (IPTG, CAS 367-93-1, Amresco, United States). The harvested DHQS cells were resuspended and sonicated in binding buffer (20 mM Tris-HCl, 0.5 M NaCl, and 5 mM imidazole, pH 8.0). The lysate was centrifuged at 10,000 × *g* for 30 min. The supernatant was loaded onto the Ni^2+^-affinity column. The protein was eluted with elution buffer (20 mM Tris-HCl, 0.5 M NaCl, 250 mM imidazole, and pH 8.0). The eluted DHQS protein was dialyzed using dialysis buffer (20 mM Tris-HCl, 100 mM NaCl, 10% glycerin, and pH 8.0) for 1 h at 4°C and stored at −80°C. Proteins concentration was determined at 595 nm using the Coomassie brilliant blue method (Yang et al., 2015).

### Construction of *aroB* Knock-Down *Mycobacterium bovis* BCG Mutant Strain

Gene silencing of the *aroB* gene in *M. bovis* BCG strain was performed, as described previously (Rock et al., 2017). To knock-down *aroB*, we designed sgRNA to target non-template strand within the open reading frame (ORF) of *aroB*. Two designed sgRNA oligonucleotides were annealed and digested with BsmBI and cloned into purified BsmBI-digested pLJR965 vector. The recombinant plasmids were transformed into *M. bovis* BCG strain, and then this strain was incubated on 7H10 medium supplemented with kanamycin. The strain was grown in 7H9 medium with or without the addition of 100 ng/ml anhydrotetracycline (ATc) to log phase, and the efficiency of CRISPRi-mediated gene silencing was detected by quantitative real-time PCR (qRT-PCR) and Western blot.

### Quantitative Real-Time PCR Assays

Briefly, the wild-type strain was grown to OD_600_ 0.6–0.8 and then treated with 0.125 and 0.25 μg/ml STR for 2, 4, 6, 12, and 24 h. The broth was collected and the RNA was extracted, as described previously (Yang et al., 2012). The cDNA was obtained using the HiScript II Q RT kit (Vazyme, Nanjing, China). The qRT-PCR system consisted of 20 μl solution containing 10 μl 2 × SYBR Green qPCR mix, specific primers, and 1 μl of cDNA. The qRT-PCR was performed on Roche 480 instrument as follows: 95°C for 1 min and 40 cycles of 95°C 15 s, 60°C 15 s, and 72°C 30 s. The expression level of each gene was normalized with *sigA* as an internal reference. Gene relative expression was determined according to the 2^–ΔΔCt^ method (Livak and Schmittgen, 2001).

### Western Blotting

*Mycobacterium* cells were sonicated and separated using 12% SDS-PAGE, and proteins were detected using an anti-DHQS antibody (Dia-An, China) at a 1:4,000 dilution. The secondary antibodies were HRP-conjugated anti-rabbit IgG (Dia-An, China). Immunoblots were visualized with a Chemiluminescence imager.

### Growth Assay and MIC

Growth assay of mycobacteria was performed. The wild-type *M. bovis* BCG strain, *aroB*-overexpressing strain (added with 30 μg/ml kan), and *aroB* knock-down mutant (100 ng/ml ATc) strain were grown to mid-log phase and then diluted to an OD_600_ of 0.1. For *aroB*-overexpressing strain, 0.125 μg/ml streptomycin was used, while for knock-down strain, 0.0625 μg/ml streptomycin was used, and 100 ng/ml ATc was added to the medium. All strains were cultured at 37°C, then sampled every day, and diluted on 7H10 solid plate for CFU counting.

The minimal inhibitory concentration (MIC) was defined as the lowest concentration of drugs that inhibited bacterial growth. The MIC was determined as described previously (Liu et al., 2016). Briefly, the *mycobacteria* were grown to the mid-log phase and diluted to ~10^5^ CFUs/ml. Then, 1 ml of the *mycobacteria* dilution was inoculated into 2 ml of Middlebrook 7H9 medium added with increasing concentrations of four anti-TB drugs and incubated at 37°C for 10 days. Then, MIC was calculated. In addition, we detected the MICs of the recombination strains to several first-line drugs using resazurin microtiter assay. The recombinant strains were grown to logarithmic stage and diluted to ~2 × 10^5^ CFUs/ml. Use 100 μl of bacterial suspension per well as the inoculum, and 100 μl of serial 2-fold dilutions of the drugs solution was added to each well of a sterile 96-well plate. The plates were subsequently sealed using parafilm and incubated at 37°C for 10 days. Then the plates were stained by adding 25 μl of 0.05% sterile resazurin solution and incubation at 37°C for another 24 h.

### Drug Affinity-Responsive Target Stability

The drug affinity response target stability (DARTS) technique was employed to analyze the interaction between DHQS and streptomycin following a previously described method (Lomenick et al., 2009; Robinson et al., 2013). Briefly, the purified His-tag fusion proteins were co-incubated with different concentrations of streptomycin at 37°C for 30 min. Then the incubated samples were digested with 6.25 μg/ml pronase E (Solarbio, CAS 9036-06-0) for 30 min at 37°C. The digestion was terminated by adding SDS-PAGE loading buffer (100 mM Tris-HCl, 4% SDS, 0.2% bromophenol blue, and 20% glycerol, pH 7.0), and digestion solution was boiled at 100°C for 10 min. Finally, the digested protein samples were detected by SDS-PAGE. The same concentration of INH and the streptomycin-unrelated protein Csm3 were used as a control.

### Isothermal Titration Calorimetry Assay

Isothermal titration calorimetry (ITC) assay was carried out to further verify the binding of DHQS to streptomycin by using a Nano ITC calorimeter (TA Instruments, New Castle, DE, United States) as described previously (Zhu et al., 2018a). Briefly, DHQS protein was dialyzed in dialysis solution (20 mM Tris-HCl, 100 mM NaCl, 10% glycerol, and pH 7.0), and streptomycin was diluted to the appropriate concentration with the dialysis solution. All the samples were degassed with a vacuum pump before loading. The protein samples were loaded into the sample cell (50 μM), and the streptomycin solution was placed into the syringe (1 mM). The parameter is set as 25 injections, 300 s interval, and stirring speed 250 r/min. The data were collected immediately and analyzed using corresponding software to obtain the *k*_d_ value. For the blank control, streptomycin titration dialysate was used in the same manner.

### Enzyme Activity Assay

Dehydroquinate synthase enzyme activity was determined using the malachite green phosphorus method, as previously described (Lambert et al., 1985; de Mendonca et al., 2011). The 60 μl reaction system contained 50 mM Tris-HCl (pH 7.5), 200 μM NAD^+^, and 200 μM DAHP. The protein and reaction solution was preheated at 37°C for 5 min, and then mixed and reacted for 30 min with the heat inactivated protein as a control. Then phosphorous reagent was added to reaction system, and reaction lasted for 30 min at 42°C to measure A820. DHQS was incubated with different concentrations of streptomycin each for 30 min, and A_820_ value was determined.

### Streptomycin Antibacterial Activity Analysis

Drug plate assay was performed to analyze the antibacterial activity of streptomycin. For the control group, the 60 μl buffer containing 50 mM Tris-HCl (pH 7.5) and 60 μM NAD^+^ was incubated with 0.25 or 0.5 mg/ml streptomycin for 30 min at 37°C. For the experimental group, 1.5 μM DHQS alone or 1.5 μM DHQS + 140 μM DAHP were added to the reaction solution and incubated with 0.25 or 0.5 mg/ml streptomycin for 30 min. All the reactions were terminated at 85°C (5 min). Then the supernatant was collected by centrifugation and added to the 7H10 plate (dilution 1:1,000), the final concentrations of streptomycin were 0.25 and 0.5 μg/ml, respectively. The wild-type *M. smegmatis* cells were grown to OD_600_ of 0.8 and then serially 10-fold diluted (from 10^−1^ to 10^−4^) in fresh 7H9 medium. Then, equal amount of culture was inoculated onto the 7H10 agar plate, and the growth of the colony was observed after 3-day incubation at 37°C.

### Liquid Chromatography Analysis

The content of streptomycin was determined through high-performance liquid chromatography (HPLC) using a Waters (Milford, MA, United States) 2695 HPLC instrument equipped with a Waters 2695 UV diode array detector (DAD), as described previously (Adams et al., 2000). The reaction system contained 60 μM NAD^+^, 0.2–1.0 mg/ml streptomycin, with or without 0.375 μM DHQS, and 140 μM DAHP in 50 mM Tris-HCl (pH 7.5). The reaction was conducted at 37°C for 30 min, followed by a 5-min termination at 85°C. The supernatant of the reaction mixture was collected by centrifugation and loaded onto a Diamonsil C18(2) column (250 × 4.6 mm, 5 μm), which was pre-equilibrated with buffer A (containing 14 mM sodium heptane sulfonate and 50 mM potassium dihydrogen phosphate) at 30°C at the flow rate of 1 ml/min. The reaction products were eluted isocratically with 12% buffer B (100% acetonitrile).

### Molecular Docking and Structural Analysis

The three-dimensional structure of DHQS in *M. tuberculosis* (PDB code: 3QBD) and that of streptomycin were downloaded from RCSB PDB database (Burley et al., 2021). Before running docking, the hydrogen atoms were added and Gasteiger charges were assigned to DHQS protein and streptomycin using AutoDockTools 1.5.7 (Morris et al., 2009). The AutoDock Vina 1.1.2 (Trott and Olson, 2010) was used for docking to predicted binding models of DHQS and streptomycin. Pymol 2.5^1^ was used for structural analysis and image production.

## Results

### DHQS Regulates Streptomycin Susceptibility

**Figure 1:**
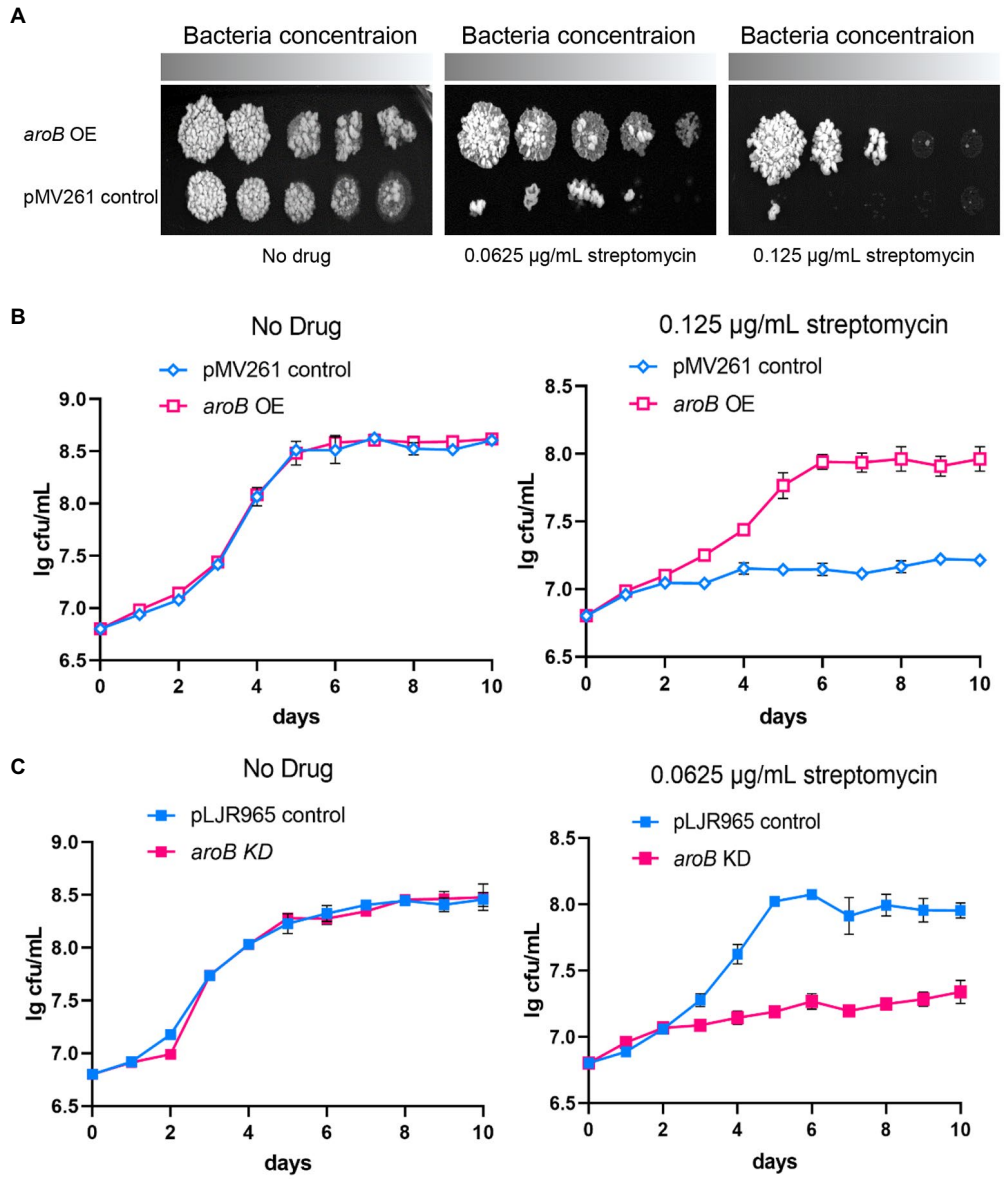
Dehydroquinate synthase (DHQS) expression level affects streptomycin susceptibility of *Mycobacterium bovis* BCG. **(A)** Overexpression of *aroB* reduced the streptomycin susceptibility of *M. bovis* BCG. The growth of *aroB* overexpressing strain and control strain (empty plasmid) on solid medium added with 0, 0.0625, and 0.125 μg/ml streptomycin was investigated. Freshly cultivated strains were grown on 7H9 medium until OD_600_ reached 1.0. The serially diluted (10^−0^, 10^−1^, 10^−2^, 10^−3^, and 10^−4^) strains were spotted onto 7H10 plates containing OADC growth supplements and cultured for another 7 days at 37°C. **(B)** Growth of *aroB* overexpressing strain and control strain in liquid medium added with 0, 0.125 μg/ml streptomycin. Growth curves of control strain and *aroB*-overexpressing strain in the medium with 0.125 μg/ml streptomycin or without drug (No drug) during 10 days. Error bars represent the SD of three biological replicates. **(C)** Knock-down of *aroB* increased the streptomycin susceptibility of *M. bovis* BCG. Aliquots were sampled at the indicated times, and the CFU was measured. Error bars represent the SD of three biological replicates.

To investigate the effect of DHQS on streptomycin susceptibility of *M. bovis* BCG, firstly, we collected bacteria from 10 days culture and detected the expression of *aroB* in *aroB*-overexpressing strain by both qRT-PCR and western blotting. As a result, the transcription level of *aroB* was upregulated by ~10-fold (Supplementary Figure 1A). And the protein expression of DHQS is significantly increased in *aroB*-overexpressing strain as well (Supplementary Figure 1B). Then we measured the growth of *aroB*-overexpressing and pMV261 control strains in the medium with or without streptomycin (Figure 1A). When serially diluted *mycobacterial* strains were spotted on the surface of solid agar medium without streptomycin, a similar bacterial colony was observed between *aroB*-overexpressing strain and control strain (Figure 1A left). When the same amount of *mycobacterial* strains were spotted on the surface of solid agar medium added with 0.0625 and 0.125 μg/ml streptomycin, a small bacterial lawn was observed for the control strain in contrast to a big lawn for *aroB*-overexpressing strain (Figure 1A middle and right). Similar results were observed when these strains were cultivated in liquid culture medium (Figure 1B). These results suggested that *aroB* potentially contributed to streptomycin susceptibility of *M. bovis*.

The MIC of streptomycin and several other anti-tuberculosis drugs against each strain were measured (Table 1; Supplementary Figure 2). The MIC of *aroB* overexpressing strain to streptomycin was 0.25 μg/ml, which was 4-fold as much as that of the control strain. In contrast, the MIC of *aroB* overexpressing strain to several other anti-TB antibiotics including isoniazid, ethambutol, and rifampicin was similar to that of control strain. In addition, we also detected the MICs of *aroB*-overexpressing strain to aminoglycoside antibiotics gentamicin and neomycin. The results showed that the MIC of gentamicin and neomycin increased by 4-fold and 64-fold, respectively. These results suggested that *aroB* mediated drug susceptibility change specific to aminoglycosides antibiotics.

**Table 1:** Minimal inhibitory concentration (MIC) of recombinant *Mycobacterium bovis* BCG strains against streptomycin and several other anti-tuberculosis drugs.

Drugs	MIC (μg/ml)	
	BCG/pMV261	BCG/pMV261-*aroB*
Streptomycin	0.0625	0.25
Gentamicin	1	4
Neomycin	2	128
Isoniazid	0.0625	0.0625
Ethambutol	8	8
Rifampicin	0.03125	0.03125

To further investigate the effect of *aroB* on streptomycin susceptibility, we constructed *aroB* knock-down strain. The downregulation of *aroB* was validated at RNA level by qPCR and protein level by western blot (Supplementary Figures 3, 4). The results showed that 100 ng/ml ATc treatment could reduce the expression of DHQS by about 5-fold and had no effect on bacterial growth after 5 days of treatment. The growth curves showed that *aroB* knock-down strain and control strain exhibited a similar growth rate in culture media without streptomycin (Figure 1C left), whereas the *aroB* knock-down strain displayed a slower growth rate than the control strain in culture media added with 0.0625 μg/ml streptomycin (Figure 1C right). The MIC of *aroB* knock-down strain to streptomycin was also measured, and results showed that the MIC decreased by 2-fold (Supplementary Figure 5). These results further confirmed that *aroB* caused streptomycin susceptibility change in *M. bovis* BCG.

### DHQS Impairs *in vitro* Antibacterial Activity of Streptomycin

**Figure 2:**
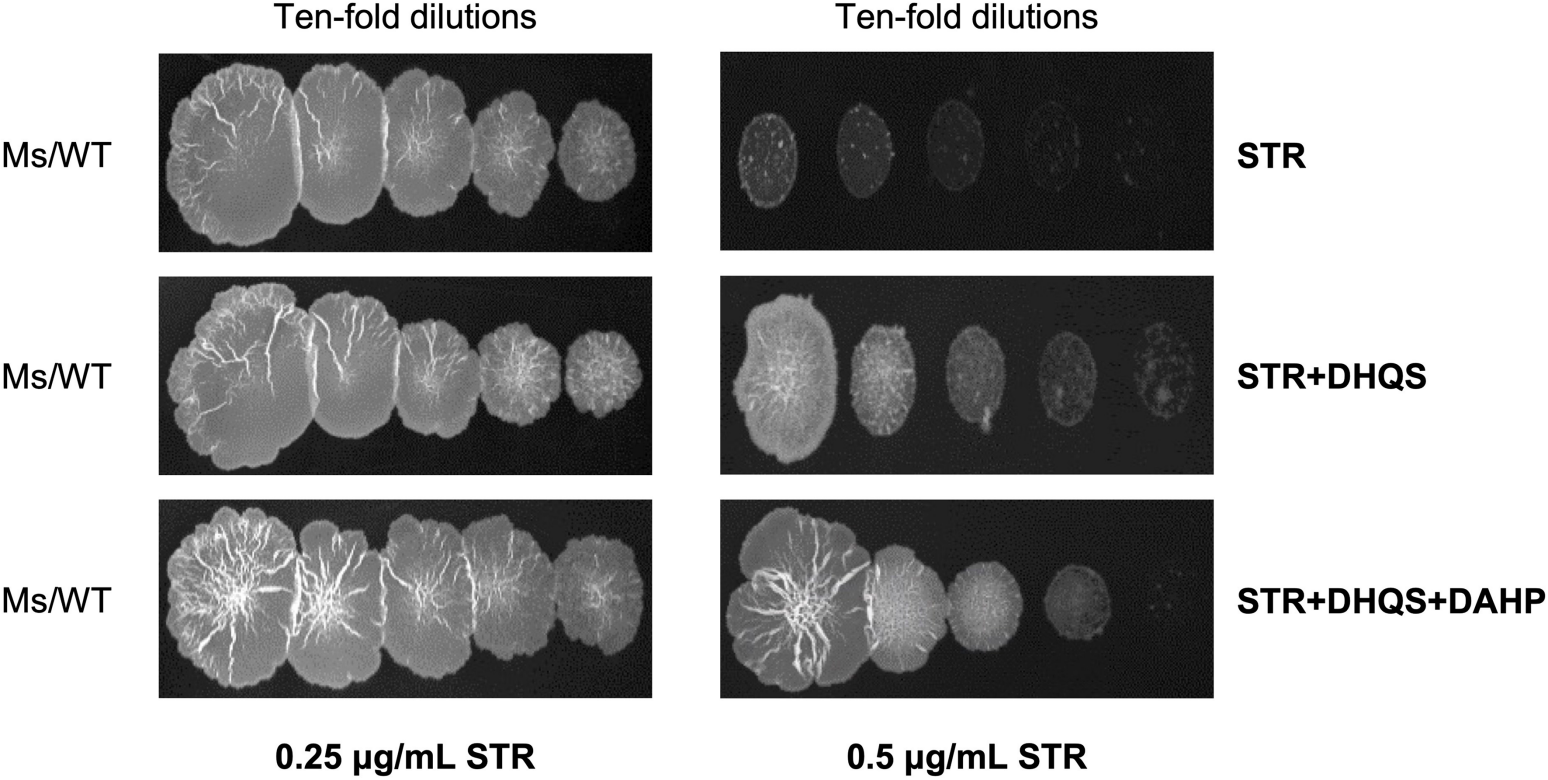
Dehydroquinate synthase impairs *in vitro* antibacterial activity of streptomycin. The 0.25 or 0.5 μg/ml streptomycin was incubated with 1.5 μM DHQS in the presence or absence of 3-deoxy-D-arabino-heptulosonate 7-phosphate (DAHP) substrate for 30 min at 37°C. The reaction mixture was added to the 7H10 plate. Wild-type *Mycobacterium smegmatis* grown to the logarithmic phase was serially diluted (10^−0^, 10^−1^, 10^−2^, 10^−3^, and 10^−4^), and equal amount of culture was spotted on the solid plate and cultivated for 3 days.

Considering that the overexpression of *aroB* reduced the streptomycin susceptibility in *M. bovis* BCG, we speculated that the enhancement mechanism of *aroB* might be related to the function of DHQS. To test this speculation, we first examined whether DHQS directly impaired the antibacterial activity of streptomycin *in vitro*. As shown in Figure 2, when the medium was added with 0.25 μg/ml streptomycin alone, *M. smegmatis* (a fast-growing model organism of *mycobacterial* species) grew well on the plate; when the medium was added with 0.25 μg/ml streptomycin in combination with 1.5 μM DHQS or 1.5 μM DHQS + 50 μM DAHP, *M. smegmatis* also grew well on the plate (Figure 2 left). However, when the medium was supplemented with 0.5 μg/ml streptomycin alone, *M. smegmatis* barely grew on the plate. In contrast, *M. smegmatis* grew well in the medium with additional 0.5 μg/ml streptomycin co-incubated with 1.5 μM DHQS or 1.5 μM DHQS + 50 μM DAHP for 30 min (Figure 2 right). These results suggested that the presence of DHQS impaired the antimicrobial activity of streptomycin against *M. smegmatis* and that the effect mediated by DHQS was due to direct interaction between streptomycin and DHQS.

We examined the streptomycin amount before and after incubation with DHQS by HPLC and found no apparent decrease in streptomycin amount (Supplementary Figure 6). This suggested susceptibility mechanism was not caused by hydrolysis or modification of streptomycin.

### DHQS Directly Binds to Streptomycin

**Figure 3:**
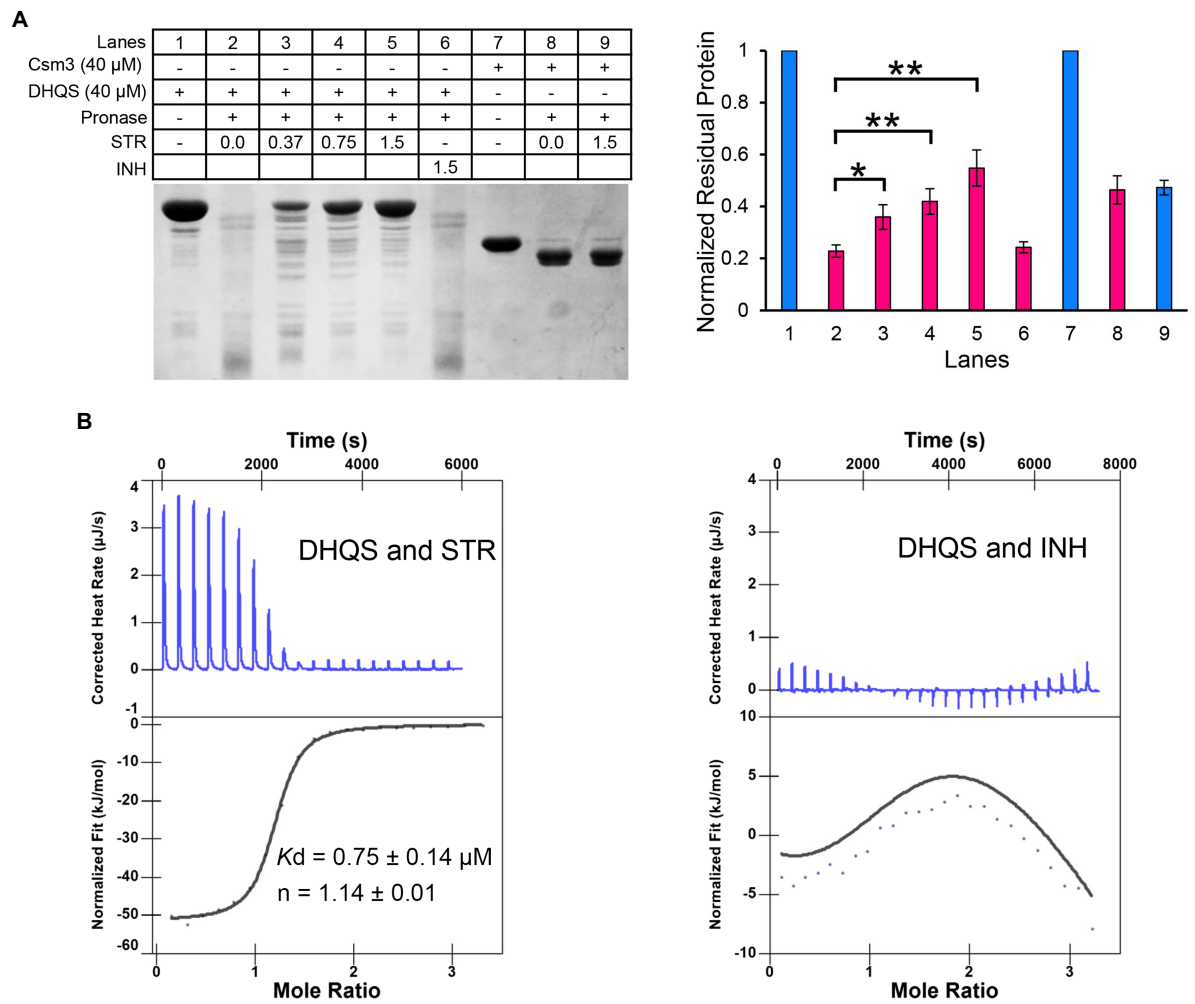
Dehydroquinate synthase directly binds to streptomycin. **(A)** Binding analysis of streptomycin and DHQS by drug affinity response target stability (DARTS). About 40 μM DHQS was incubated with increasing concentrations of streptomycin (0.37, 0.75, and 1.5 mM) or 1.5 mM INH (negative control) for 30 min at 37°C, followed by digestion with 180 ng of pronase E for 30 min at 37°C. All the samples were subjected to a 12% w/v SDS-PAGE. The amount of protein degradation at each concentration was quantified with the non-digested lane as internal reference. Barplot shows the mean and SD of three biological replicates. *p*-values were calculated by GraphPad Prism 8 with an unpaired two-tailed Student’s *t*-test. Asterisk represents the significant difference between the two groups of data (^*^p < 0.05, ^**^p < 0.01). **(B)** Isothermal titration calorimetry (ITC) analysis of 50 μM DHQS binding with 1 mM streptomycin (left) or 1 mM isoniazid (right). Original titration data and integrated heat measurements are shown in the upper and lower panel, respectively. The solid line in the left lower panel represents the best fitting of a single-site binding model of DHQS and streptomycin, while right lower panel shows no interaction between DHQS and isoniazid.

The DARTS method was employed to detect the binding of streptomycin to DHQS. DHQS alone was digested by pronase E with ~20% DHQS remaining undigested, whereas the proteolysis of DHQS was decreased with maximum remaining amount of ~55% when the co-incubated streptomycin was increased from 0.37 mM to 1.5 mM, indicating that streptomycin protected DHQS from degradation (Figure 3A). In contrast, isoniazid showed no protection effect on DHQS. In addition, no protection effect of streptomycin on the control protein Csm3 was observed (Figure 3A). These results suggested that streptomycin specifically interacted with DHQS to increase the stability of DHQS (resistance of DHQS to proteolysis).

We have further validated the direct interaction between DHQS and streptomycin by the ITC (Figure 3B). The binding stoichiometry between streptomycin and DHQS was 1:1 (*n* = 1.14). The binding affinity was *K*_d_ = 0.75 ± 0.14 μM. In contrast, no specific binding of INH with DHQS was observed under the same conditions.

Taken together, both DARTS and ITC assays suggested specific binding of DHQS to streptomycin.

### Binding Site and Binding Model of DHQS and Streptomycin

To further explore the binding mechanism, we first investigated the potential binding site of streptomycin. There are two known binding sites in DHQS, namely, the NAD^+^ co-factor and the DAHP substrate binding pockets (Supplementary Figure 7). Because these two pockets are both essential for the catalytic activity of DHQS, the binding of the streptomycin to either of these two sites is expected to competitively disturb DHQS activity. *In vitro* DHQS enzyme activity assay was conducted under the increasing concentrations of streptomycin. Unexpectedly, the results indicated that streptomycin had no apparent influence on the catalytic activity of DHQS (Supplementary Figure 8). This suggested that streptomycin bound to neither the NAD^+^ binding site nor DAHP binding site and that DHQS-mediated streptomycin susceptibility in a catalytic activity-independent manner. Based on these results, we speculated that there should be at least one alternative site in DHQS for streptomycin binding.

**Figure 4:**
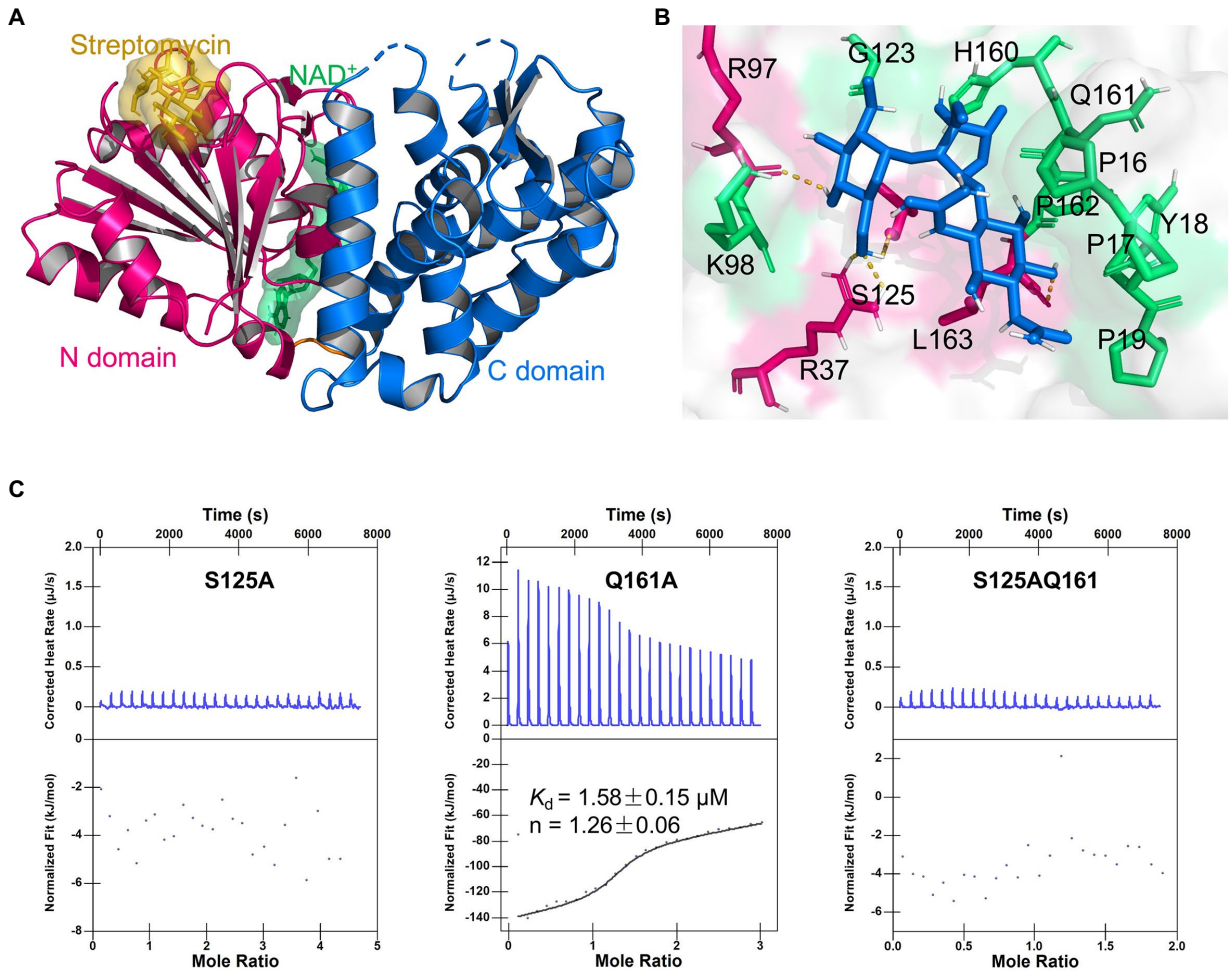
Binding model of DHQS and streptomycin. **(A)** Streptomycin binds to the proposed site of DHQS. **(B)** Residues involved in the binding of streptomycin and DHQS. **(C)** ITC analysis of 1 mM streptomycin binding to 50 μM mutant proteins (S125A, Q161A, and S125AQ161A). ITC was carried out with 1 mM streptomycin and mutant protein concentration of 50 μM.

Further, we examined the surface of the DHQS and identified a novel binding site for streptomycin. This site was located at the N-terminal of DHQS, and it was close to DHQS dimer interface. Particularly, this site was located behind but spatially apart from the binding sites of NAD^+^ and substrate to DHQS (Supplementary Figure 9). We then modeled the binding form of streptomycin to this site using molecular docking. In this model, streptomycin bound to the N-terminal domain of DHQS (Figure 4A; Supplementary Figure 10). The residues around streptomycin included P16, P17, Y18, P19, R37, R97, K98, G123, S125, H160, Q161, P162, and L163. Especially, residue P17, R37, R97, and S125 formed hydrogen bonds with streptomycin (Figure 4B). The model also supported the 1:1 binding of streptomycin and DHQS (Supplementary Figure 10).

To validate the proposed binding site for streptomycin, we constructed S125A, Q161A, and S125AQ161A mutant proteins using site-directed mutagenesis and tested their binding with streptomycin (Figure 4C). The ITC assay indicated that the S125A mutant lost the streptomycin binding ability. The Q161A mutant shown a weaker streptomycin binding ability (*K*_d_ = 1.58 ± 0.15 μM) than WT DHQS (*K*_d_ = 0.75 ± 0.14 μM). This difference might be attributed to the fact that hydrogen bond interaction energy was higher than van der Waals interaction energy. As expected, the S125AQ161A double mutant also lost the streptomycin binding ability. In addition, DARTS analysis also showed that streptomycin lost protection against protease cleavage in S125A mutants (Supplementary Figure 11). The MIC of streptomycin against *aroB* S125A OE and Q161A OE strains was also measured. The results showed that MIC of streptomycin against *aroB* Q161A OE strain was 4-fold higher than that of control strain and same as that of *aroB*-overexpressing strain, while there was no significant effect of streptomycin on *in vitro* growth of BCG expressing the aroB S125A and the control strain (Supplementary Figure 12). These results indicated that streptomycin was very likely to bind to the proposed site and that S125 was an key residue for streptomycin binding.

### Streptomycin Induces DHQS Expression

**Figure 5:**
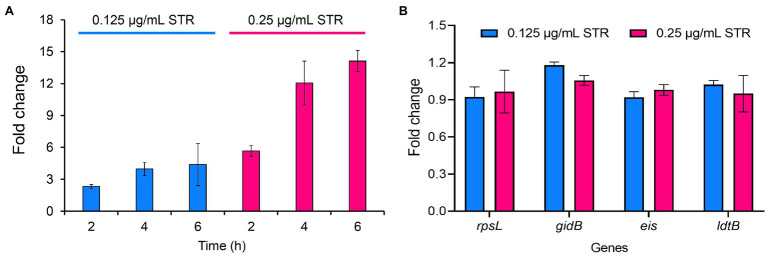
Streptomycin induces increased expression of *aroB* in *Mycobacterium bovis* BCG. **(A)** Expression of *aroB* at 2, 4, 6 h post-cultivation. The mid-log phase of *M. bovis* BCG (OD_600_ = 1.0) was cultivated with 0.125 and 0.25 μg/ml streptomycin to induce the expression of *aroB*, and *aroB* expression in *M. bovis* BCG strain was detected by quantitative real-time PCR (qRT-PCR). **(B)** Expression of *rpsL*, *gidB*, *eis*, and *ldtB* genes at 6 h post-cultivation. The expression of streptomycin resistance-related genes *rpsL* and *gidB*, and unrelated genes *eis* and *ldtB* after streptomycin treatment were quantitatively detected. Relative expression levels of the genes were normalized with the *sigA* gene as internal reference. The data were analyzed using the 2^−ΔΔCt^ method. Barplot shows the mean and SD of three biological replicates.

To explore the expression of DHQS under streptomycin treatment, we performed qRT-PCR assay. The expression of *aroB* exhibited a 2.3-fold and 5.7-fold increase at 2 h post-cultivation of BCG strains with 0.125 and 0.25 μg/ml streptomycin, a 4.0-fold and 12.1-fold increase at 4 h, and a 4.4-fold and 14.1-fold increase at 6 h, respectively (Figure 5A). In contrast, streptomycin treatment had a limited influence on several other control genes (Figure 5B). The results indicated that streptomycin specifically induced the expression of *aroB* at the RNA level.

## Discussion

### Alternative Function of DHQS in Streptomycin Susceptibility

Streptomycin is the first discovered effective anti-TB antibiotic and remains to be one of the essential first-line anti-TB drugs. In the current study, genetics analysis indicated that DHQS regulates the susceptibility of *M. bovis* BCG to streptomycin. In addition, we have checked several genome re-sequencing studies of the drug-resistant clinical isolates of the *M. tuberculosis* (Desjardins et al., 2016; Manson et al., 2017; Coll et al., 2018). None of these studies reports the mutation of *aroB* in streptomycin resistance isolates. Together with our findings in current study, these results indicate *aroB* contributes to the intrinsic resistance, other than acquired resistance that usually caused by gene mutation. Therefore, DHQS represents a new intrinsic determinant for streptomycin susceptibility of *M. bovis* BCG. Apart from the classic function of DHQS in shikimic acid pathway (Carpenter et al., 1998), this study for the first time reports the alternative function of DHQS in antimicrobial susceptibility. *In vitro* biochemistry assay indicates that streptomycin shows no inhibition on dehydroquinate synthase activity of DHQS, suggesting that DHQS-mediated susceptibility is independent from the shikimic acid pathway. Since *M. bovis* BCG and *M. tuberculosis* exhibit 100% DHQS sequence identity, we speculate that DHQS plays the same role in *M. tuberculosis* as in *M. bovis* BCG. Targeting intrinsic resistance genes can increase or restore the susceptibility of bacteria to existing antibiotics (Blair et al., 2015). Therefore, the streptomycin in combination of the inhibitors targeting DHQS may be a potential strategy for TB treatment.

### Mechanisms by Which DHQS-Mediated Streptomycin Susceptibility

**Figure 6:**
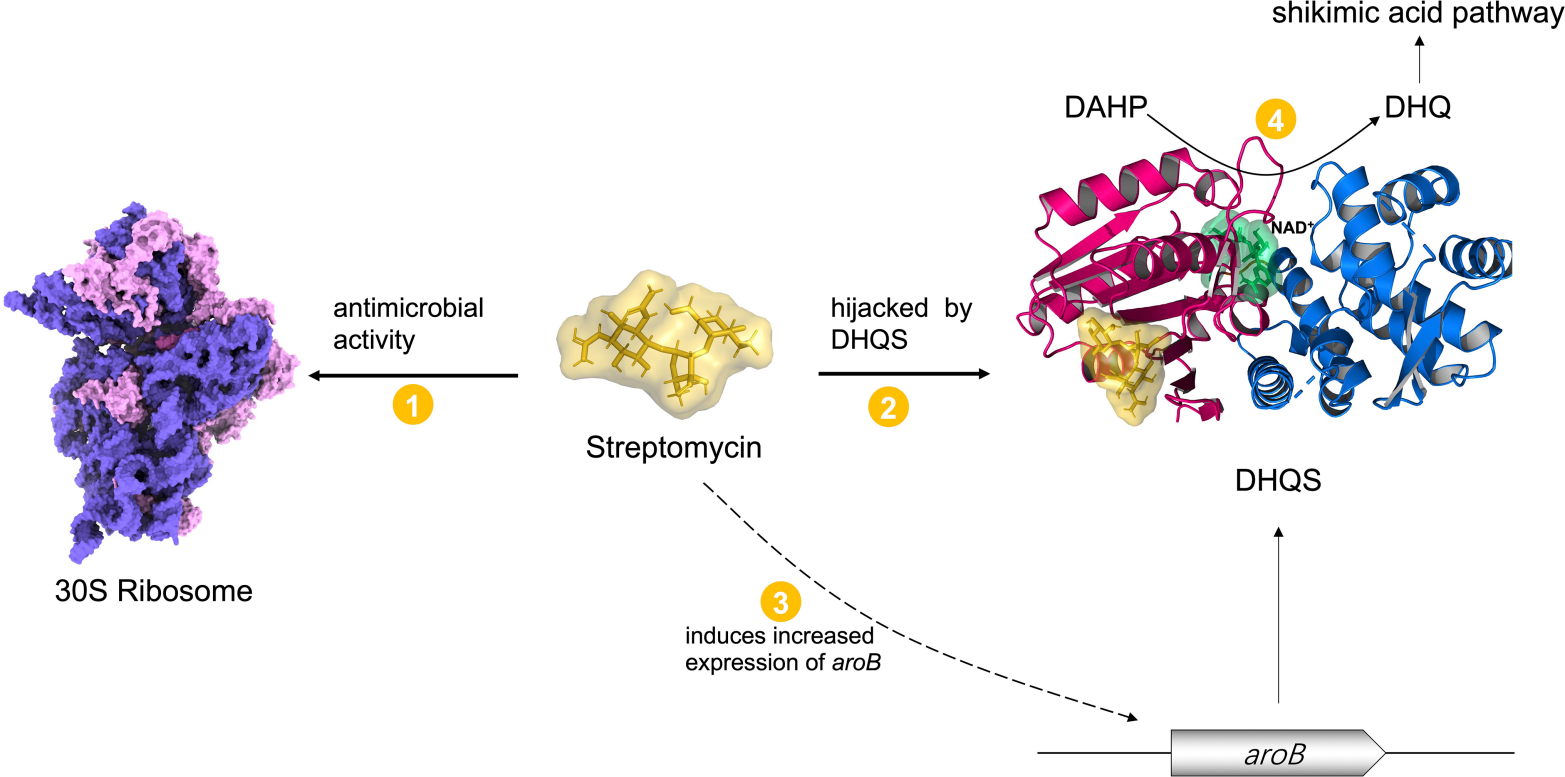
A model in which DHQS mediates streptomycin susceptibility. Streptomycin inhibits bacteria by targeting 30S ribosome. The expression of DHQS protein was increased under streptomycin treatment. DHQS directly bound to streptomycin while retaining its own normal catalytic activity. As a result, DHQS “hijacks” streptomycin, thus reducing the binding of streptomycin to 30S ribosome target, eventually reducing the susceptibility of bacteria to streptomycin.

The several antibiotics resistance mechanisms have been reported, including mutation of the target, reduction of cell permeability, antibiotic efflux, expression changes of the target, target protection, and modification/degradation of antibiotic (Boolchandani et al., 2019). Streptomycin kills bacteria by binding bacterial 30S ribosome to interfere with protein synthesis. Mutation of 30S ribosome-related genes *rrs* and *rpsL* can cause the acquired streptomycin resistance. In addition, mutation of *gidB* modifying 30S ribosome can also result in acquired streptomycin resistance (Coll et al., 2018). In some other pathogens, the genes involved in encoding streptomycin modification enzymes, such as aminoglycoside phosphotransferases and aminoglycoside acetyltransferases, contribute to streptomycin resistance (Morar and Wright, 2010). Currently, we find that DHQS regulates antibiotic susceptibility in a non-canonical way. Our biochemical analysis indicated that streptomycin directly bound to DHQS. The accurate binding model of DHQS and streptomycin remains to be further investigated *via* co-crystallization. At present, we employed biochemical assays and computational modeling to explore the binding mode. First, DHQS contains two known ligand binding sites, including NAD^+^ co-factor and the substrate DAHP binding pockets. However, these two binding sites are excluded as our experimental analysis indicated that streptomycin does not impact the catalytic activity of DHQS. Therefore, we tried to identify candidate site by artificial manner. We visually inspected the surface structure of DHQS to search the candidate pockets that show potential to accommodate the size of streptomycin molecule and keep distance away from NAD^+^ and the DAHP binding pockets. The 1:1 binding of streptomycin and DHQS from our ITC assay was also taken into consideration. Based on these restraints, a candidate pocket was identified. We further used computational modeling and mutation assay to validate the candidate pocket. In this case, we selected the binding mode with lowest RMSD value and showed in Figure 4B and Supplementary Figure 10. And the two representative residues, S125 and Q161 were selected for mutation analysis. Interestingly, S125A mutant of DHQS completely lost binding affinity to streptomycin, suggesting that streptomycin was very likely to bind at our proposed site, and we named it streptomycin “hijack” site. Based on these results, we proposed a candidate model in which DHQS-mediated streptomycin resistance (Figure 6). In the proposed model, DHQS directly bound to streptomycin at the “hijack” site, thus reducing the attack of streptomycin to its main antibacterial target 30S ribosome. Therefore, DHQS acted as a neutralizer for streptomycin toxicity. It should be noted that in this model, DHQS retained its fundamental catalytic activity in the shikimic acid pathway. Compared with known AMR models (Boolchandani et al., 2019; Wilson et al., 2020), our model provides an insight into a non-canonical resistance mechanism by which protein hijacked antibiotic to reduce antibiotic–target interaction while maintaining its own normal enzyme function.

### Potential DHQS Regulation Pathway Under Streptomycin Induction

In our proposed model, the effect of streptomycin toxicity neutralization was dependent on the expression level of DHQS. In the current study, we found that streptomycin induced expression of DHQS, in turn enhancing DHQS-mediated neutralization of streptomycin toxicity to bacteria. Our results revealed a positive feedback mechanism by which bacteria increased DHQS expression in response to streptomycin stress. However, the pathway in which streptomycin induces DHQS remains largely unclear. To this end, transcriptional regulators responding to streptomycin are suggested to be further studied. Transcriptional regulator WhiB7 can be induced by streptomycin to regulate intrinsic antibiotic resistance of *M. tuberculosis* (Morris et al., 2005). Multiple antibiotic resistance repressor (MarR) family regulators can recognize antibiotics including streptomycin and activate target genes expression, thus conferring bacteria antibiotics resistance (Chang et al., 2013; Gagneux, 2018). *Mycobacterium tuberculosis* genome encodes eight MarR-like regulators (Gao et al., 2017). It will be worthwhile to investigate whether these regulators interact with streptomycin and participate in the regulation of DHQS expression in the future.

## Data Availability Statement

The original contributions presented in the study are included in the article/**Supplementary Material**, further inquiries can be directed to the corresponding authors.

## Author Contributions

WW, JQ, XJ, LC, and XH completed the experiments. WW and TC conducted the experiments and data analysis. WW, MY, and TC wrote the manuscript. JH, MC, MY, and TC revised the manuscript. All authors contributed to the article and approved the submitted version.

## Funding

This work was supported by the Science, Technology, and Innovation Commission of Shenzhen Municipality (JCYJ20180306171604974), the Fundamental Research Funds for the Central Universities (3102020smxy002), the National Natural Science Foundation of China (32070079), and the Fundamental Research Funds for the Central Universities (2020kfyXJJS119).

## Conflict of Interest

The authors declare that the research was conducted in the absence of any commercial or financial relationships that could be construed as a potential conflict of interest.

## Publisher’s Note

All claims expressed in this article are solely those of the authors and do not necessarily represent those of their affiliated organizations, or those of the publisher, the editors and the reviewers. Any product that may be evaluated in this article, or claim that may be made by its manufacturer, is not guaranteed or endorsed by the publisher.

## Footnotes

^1^ https://pymol.org/

## References

[ref1] Adams E. Rafiee M. Roets E. Hoogmartens J. (2000). Liquid chromatographic analysis of streptomycin sulfate. J. Pharm. Biomed. Anal. 24, 219–226. doi: 10.1016/s0731-7085(00)00413-1, PMID: 11130201

[ref2] Aldridge B. B. Barros-Aguirre D. Barry C. E. Bates R. H. Berthel S. J. Boshoff H. I.. (2021). The tuberculosis drug accelerator at year 10: what have we learned? Nat. Med. 27, 1333–1337. doi: 10.1038/s41591-021-01442-2, PMID: 34226736 PMC10478072

[ref3] Alekseeva M. G. Boyko K. M. Nikolaeva A. Y. Mavletova D. A. Rudakova N. N. Zakharevich N. V.. (2019). Identification, functional and structural characterization of novel aminoglycoside phosphotransferase APH(3″)-Id from *Streptomyces rimosus* subsp. rimosus ATCC 10970. Arch. Biochem. Biophys. 671, 111–122. doi: 10.1016/j.abb.2019.06.008, PMID: 31251922

[ref4] Blair J. M. Webber M. A. Baylay A. J. Ogbolu D. O. Piddock L. J. (2015). Molecular mechanisms of antibiotic resistance. Nat. Rev. Microbiol. 13, 42–51. doi: 10.1038/nrmicro3380 25435309

[ref5] Boolchandani M. D’Souza A. W. Dantas G. (2019). Sequencing-based methods and resources to study antimicrobial resistance. Nat. Rev. Genet. 20, 356–370. doi: 10.1038/s41576-019-0108-4, PMID: 30886350 PMC6525649

[ref6] Burley S. K. Bhikadiya C. Bi C. Bittrich S. Chen L. Crichlow G. V.. (2021). RCSB protein data bank: powerful new tools for exploring 3D structures of biological macromolecules for basic and applied research and education in fundamental biology, biomedicine, biotechnology, bioengineering and energy sciences. Nucleic Acids Res. 49, D437–D451. doi: 10.1093/nar/gkaa1038, PMID: 33211854 PMC7779003

[ref7] Carpenter E. P. Hawkins A. R. Frost J. W. Brown K. A. (1998). Structure of dehydroquinate synthase reveals an active site capable of multistep catalysis. Nature 394, 299–302. doi: 10.1038/28431, PMID: 9685163

[ref8] Chang Y. M. Chen C. K. Ko T. P. Chang-Chien M. W. Wang A. H. (2013). Structural analysis of the antibiotic-recognition mechanism of MarR proteins. Acta Crystallogr. D Biol. Crystallogr. 69, 1138–1149. doi: 10.1107/s0907444913007117, PMID: 23695258

[ref9] Coll F. Phelan J. Hill-Cawthorne G. A. Nair M. B. Mallard K. Ali S.. (2018). Genome-wide analysis of multi- and extensively drug-resistant *Mycobacterium tuberculosis*. Nat. Genet. 50, 307–316. doi: 10.1038/s41588-017-0029-0, PMID: 29358649

[ref10] de Mendonca J. D. Adachi O. Rosado L. A. Ducati R. G. Santos D. S. Basso L. A. (2011). Kinetic mechanism determination and analysis of metal requirement of dehydroquinate synthase from *Mycobacterium tuberculosis* H37Rv: an essential step in the function-based rational design of anti-TB drugs. Mol. BioSyst. 7, 119–128. doi: 10.1039/c0mb00085j, PMID: 20978656

[ref11] de Mendonça J. D. Ely F. Palma M. S. Frazzon J. Basso L. A. Santos D. S. (2007). Functional characterization by genetic complementation of aroB-encoded dehydroquinate synthase from *Mycobacterium tuberculosis* H37Rv and its heterologous expression and purification. J. Bacteriol. 189, 6246–6252. doi: 10.1128/jb.00425-07, PMID: 17586643 PMC1951933

[ref12] Desjardins C. A. Cohen K. A. Munsamy V. Abeel T. Maharaj K. Walker B. J.. (2016). Genomic and functional analyses of *Mycobacterium tuberculosis* strains implicate ald in D-cycloserine resistance. Nat. Genet. 48, 544–551. doi: 10.1038/ng.3548, PMID: 27064254 PMC4848111

[ref13] Gagneux S. (2018). Ecology and evolution of *Mycobacterium tuberculosis*. Nat. Rev. Microbiol. 16, 202–213. doi: 10.1038/nrmicro.2018.8, PMID: 29456241

[ref14] Gao Y. R. Li D. F. Fleming J. Zhou Y. F. Liu Y. Deng J. Y.. (2017). Structural analysis of the regulatory mechanism of MarR protein Rv2887 in *M. tuberculosis*. Sci. Rep. 7:6471. doi: 10.1038/s41598-017-01705-4, PMID: 28743871 PMC5526998

[ref15] Lambert J. M. Boocock M. R. Coggins J. R. (1985). The 3-dehydroquinate synthase activity of the pentafunctional arom enzyme complex of *Neurospora crassa* is Zn2+−dependent. Biochem. J. 226, 817–829. doi: 10.1042/bj2260817, PMID: 3157372 PMC1144782

[ref16] Lewis K. (2013). Platforms for antibiotic discovery. Nat. Rev. Drug Discov. 12, 371–387. doi: 10.1038/nrd3975, PMID: 23629505

[ref17] Liu J. S. Cheng W. C. Wang H. J. Chen Y. C. Wang W. C. (2008). Structure-based inhibitor discovery of *Helicobacter pylori* dehydroquinate synthase. Biochem. Biophys. Res. Commun. 373, 1–7. doi: 10.1016/j.bbrc.2008.05.070, PMID: 18503755

[ref18] Liu Y. Wang H. Cui T. Zhou X. Jia Y. Zhang H.. (2016). NapM, a new nucleoid-associated protein, broadly regulates gene expression and affects mycobacterial resistance to anti-tuberculosis drugs. Mol. Microbiol. 101, 167–181. doi: 10.1111/mmi.13383, PMID: 27010232

[ref19] Livak K. J. Schmittgen T. D. (2001). Analysis of relative gene expression data using real-time quantitative PCR and the 2(-Delta Delta C(T)) method. Methods 25, 402–408. doi: 10.1006/meth.2001.1262, PMID: 11846609

[ref20] Lomenick B. Hao R. Jonai N. Chin R. M. Aghajan M. Warburton S.. (2009). Target identification using drug affinity responsive target stability (DARTS). Proc. Natl. Acad. Sci. U. S. A. 106, 21984–21989. doi: 10.1073/pnas.0910040106, PMID: 19995983 PMC2789755

[ref21] Manson A. L. Cohen K. A. Abeel T. Desjardins C. A. Armstrong D. T. Barry C. E.. (2017). Genomic analysis of globally diverse *Mycobacterium tuberculosis* strains provides insights into the emergence and spread of multidrug resistance. Nat. Genet. 49, 395–402. doi: 10.1038/ng.3767, PMID: 28092681 PMC5402762

[ref22] Morar M. Wright G. D. (2010). The genomic enzymology of antibiotic resistance. Annu. Rev. Genet. 44, 25–51. doi: 10.1146/annurev-genet-102209-163517, PMID: 20822442

[ref23] Morris G. M. Huey R. Lindstrom W. Sanner M. F. Belew R. K. Goodsell D. S.. (2009). AutoDock4 and AutoDockTools4: automated docking with selective receptor flexibility. J. Comput. Chem. 30, 2785–2791. doi: 10.1002/jcc.21256, PMID: 19399780 PMC2760638

[ref24] Morris R. P. Nguyen L. Gatfield J. Visconti K. Nguyen K. Schnappinger D.. (2005). Ancestral antibiotic resistance in *Mycobacterium tuberculosis*. Proc. Natl. Acad. Sci. U. S. A. 102, 12200–12205. doi: 10.1073/pnas.0505446102, PMID: 16103351 PMC1186028

[ref25] Neetu N. Katiki M. Dev A. Gaur S. Tomar S. Kumar P. (2020). Structural and biochemical analyses reveal that chlorogenic acid inhibits the shikimate pathway. J. Bacteriol. 202, e00248–e00320. doi: 10.1128/jb.00248-20, PMID: 32661075 PMC7925078

[ref26] Nichols C. E. Ren J. Lamb H. K. Hawkins A. R. Stammers D. K. (2003). Ligand-induced conformational changes and a mechanism for domain closure in *Aspergillus nidulans* dehydroquinate synthase. J. Mol. Biol. 327, 129–144. doi: 10.1016/s0022-2836(03)00086-x, PMID: 12614613

[ref27] Okamoto S. Tamaru A. Nakajima C. Nishimura K. Tanaka Y. Tokuyama S.. (2007). Loss of a conserved 7-methylguanosine modification in 16S rRNA confers low-level streptomycin resistance in bacteria. Mol. Microbiol. 63, 1096–1106. doi: 10.1111/j.1365-2958.2006.05585.x, PMID: 17238915

[ref28] Pai M. Behr M. A. Dowdy D. Dheda K. Divangahi M. Boehme C. C.. (2016). Tuberculosis. Nat. Rev. Dis. Primers 2:16076. doi: 10.1038/nrdp.2016.76 27784885

[ref29] Poehlsgaard J. Douthwaite S. (2005). The bacterial ribosome as a target for antibiotics. Nat. Rev. Microbiol. 3, 870–881. doi: 10.1038/nrmicro1265, PMID: 16261170

[ref30] Robinson T. J. Pai M. Liu J. C. Vizeacoumar F. Sun T. Egan S. E.. (2013). High-throughput screen identifies disulfiram as a potential therapeutic for triple-negative breast cancer cells: interaction with IQ motif-containing factors. Cell Cycle 12, 3013–3024. doi: 10.4161/cc.26063, PMID: 23974104 PMC3875676

[ref31] Rock J. M. Hopkins F. F. Chavez A. Diallo M. Chase M. R. Gerrick E. R.. (2017). Programmable transcriptional repression in mycobacteria using an orthogonal CRISPR interference platform. Nat. Microbiol. 2:16274. doi: 10.1038/nmicrobiol.2016.274, PMID: 28165460 PMC5302332

[ref32] Singh V. Chibale K. (2021). Strategies to combat multi-drug resistance in tuberculosis. Acc. Chem. Res. 54, 2361–2376. doi: 10.1021/acs.accounts.0c00878, PMID: 33886255 PMC8154215

[ref33] Stern A. L. Van der Verren S. E. Kanchugal P. S. Näsvall J. Gutiérrez-de-Terán H. Selmer M. (2018). Structural mechanism of AadA, a dual-specificity aminoglycoside adenylyltransferase from *Salmonella enterica*. J. Biol. Chem. 293, 11481–11490. doi: 10.1074/jbc.RA118.003989, PMID: 29871922 PMC6065190

[ref34] Trott O. Olson A. J. (2010). AutoDock Vina: improving the speed and accuracy of docking with a new scoring function, efficient optimization, and multithreading. J. Comput. Chem. 31, 455–461. doi: 10.1002/jcc.21334, PMID: 19499576 PMC3041641

[ref35] Wilson D. N. (2014). Ribosome-targeting antibiotics and mechanisms of bacterial resistance. Nat. Rev. Microbiol. 12, 35–48. doi: 10.1038/nrmicro3155, PMID: 24336183

[ref36] Wilson D. N. Hauryliuk V. Atkinson G. C. O’Neill A. J. (2020). Target protection as a key antibiotic resistance mechanism. Nat. Rev. Microbiol. 18, 637–648. doi: 10.1038/s41579-020-0386-z, PMID: 32587401

[ref37] Wright G. D. (2007). The antibiotic resistome: the nexus of chemical and genetic diversity. Nat. Rev. Microbiol. 5, 175–186. doi: 10.1038/nrmicro1614, PMID: 17277795

[ref38] Yang M. Gao C. Cui T. An J. He Z. G. (2012). A TetR-like regulator broadly affects the expressions of diverse genes in *Mycobacterium smegmatis*. Nucleic Acids Res. 40, 1009–1020. doi: 10.1093/nar/gkr830, PMID: 21976733 PMC3273814

[ref39] Yang M. Gao C. H. Hu J. Zhao L. Huang Q. He Z. G. (2015). InbR, a TetR family regulator, binds with isoniazid and influences multidrug resistance in *Mycobacterium bovis* BCG. Sci. Rep. 5:13969. doi: 10.1038/srep13969, PMID: 26353937 PMC4564863

[ref40] Zhang H. Li D. Zhao L. Fleming J. Lin N. Wang T.. (2013). Genome sequencing of 161 *Mycobacterium tuberculosis* isolates from China identifies genes and intergenic regions associated with drug resistance. Nat. Genet. 45, 1255–1260. doi: 10.1038/ng.2735, PMID: 23995137

[ref41] Zhu C. Liu Y. Hu L. Yang M. (2018a). Molecular mechanism of the synergistic activity of ethambutol and isoniazid against *Mycobacterium tuberculosis*. J. Biol. Chem. 293, 16741–16750. doi: 10.1074/jbc.RA118.002693, PMID: 30185616 PMC6204910

[ref42] Zhu N. Wang X. Li D. Lin Y. You X. Jiang J.. (2018b). IMB-T130 targets 3-dehydroquinate synthase and inhibits *Mycobacterium tuberculosis*. Sci. Rep. 8:17439. doi: 10.1038/s41598-018-35701-z, PMID: 30487577 PMC6262012

