# Supplementary material for: Dehydroquinate Synthase Directly Binds to Streptomycin and Regulates Susceptibility of Mycobacterium bovis to Streptomycin in a Non-canonical Mode
Source: Front Microbiol. 2022 Apr 19;13:818881. doi: 10.3389/fmicb.2022.818881 (PMC9063660; doi:10.3389/fmicb.2022.818881)
Supplement: Supplementary file 1 [file Data_Sheet_1.pdf]

Supplementary Materials

Supplementary Table 1. Bacterial strains and plasmids used in this study.

**Strains and plasmids**	**Relevant genotype or features**	**Source**
**Strain**		
E.coli		
DH5α	Host for plasmid construction	Novagen
BL21	Host for overexpression	Novagen
M. smegmatis MC^2 155	*M. smegmatis* MC^2 155 wild type	ATCC
M. bovis BCG	*M. bovis* BCG wild type	ATCC
BCG/pMV261	BCG with pMV261	This study
BCG/pMV261-*aroB*	*aroB*-overexpressing strain	This study
BCG/pLJR965-*aroB*	*aroB* knockdown strain	This study
**Plasmid**		
pET28a	Kan^r, *lacZ* operon, T7 promotor, N-termial His-Tag	Novagen
pET28a-*aroB*	*aroB* inserted in *EcoR*I-*Xba*I of pET28a	This study
pET28a-*aroB*(E75A)	*aroB*(E75A) inserted in *EcoR*I-*Xba*I of pET28a	This study
pET28a-*aroB*(D111G)	*aroB*(D111G) inserted in *EcoR*I-*Xba*I of pET28a	This study
pET28a-*aroB*(E256A)	*aroB*(E256A) inserted in *EcoR*I-*Xba*I of pET28a	This study
pET28a-*aroB*(S125A)	*aroB*(S125A) inserted in *EcoR*I-*Xba*I of pET28a	This study
pET28a-*aroB*(Q161A)	*aroB*(Q161A) inserted in *EcoR*I-*Xba*I of pET28a	This study
pMV261	Kan^r, pAL5000 replicon	This study
pMV261-*aroB*	*aroB* inserted in *EcoR*I-*Xba*I of pMV261	This study
pLJR965	Kan^r, TetR promoter, CRISPRi vector	Rock et al., 2017
pLJR965-*aroB* sgRNA	*aroB* sgRNA inserted in *BsmB*I of pLJR965	This study

Supplementary Table 2. Primers used in this study.

Name	Sequence (5’-3’)	Note
AroB-F	ATAA**GAATTC**ATGACCGATATCGGCGCAC (*EcoR*I)	Clone and expression
AroB-R	CAGT**TCTAGA**TCATGGGGCGCAAACTC (*Xba*I)	Clone and expression
AroB(E75A)-F	ACGCCAAGGCCGGCAAGGACCTGCCCGTCGTGGGATT	Mutagenesis
AroB(E75A)-R	CTTGCCGGCTTCGGCGTCGGGGATCTCGATGCGGTGC	Mutagenesis
AroB(D111G)-F	ACGCGATCAAGCGCCGGGAACGCTACCGGTGGCGCCA	Mutagenesis
AroB(D111G)-R	TTCCCGGCGTTCGATCGCGTGGCCTAATGTGTGGCCG	Mutagenesis
AroB(E256A)-F	CACGCGATCGCGCGCCGGGAACGCTACCGGTGGCGCCACGGC	Mutagenesis
AroB(E256A)-R	TTCCCGGCGCGCGATCGCGTGGCCTAATGTGTGGCCGTAGTT	Mutagenesis
AroB(S125A)-F	GCGTCGCGATTGTGCACCTGC	Mutagenesis
AroB(S125A)-R	GCAGGTGCACAATCGCGACGC	Mutagenesis
AroB(Q161A)-F	GGCGTTTCATGCGCCGTTGGC	Mutagenesis
AroB(Q161A)-R	GCCAACGGCGCATGAAACGCC	Mutagenesis
aroB sgRNA-F	GGGAGCTGGAAATCATGGCCGGCGA	Primer, clone to pLJR965
aroB sgRNA-R	AAACTCGCCGGCCATGATTTCCAGC	Primer, clone to pLJR965
aroB RT-F	CTGGGCATGGTCGATGCGGCC	qRT-PCR
aroB RT-R	GGCCTTGACCACTTCGGCCAT	qRT-PCR
eis RT-F	GCTACCACCTTGCATGAGCTG	qRT-PCR
eis RT-R	CAGCCCGCCCGGCACCTGCTG	qRT-PCR
rpsL RT-F	GAAGGTTGCCCGCGTGAAGTT	qRT-PCR
rpsL RT-R	TATCCAGCGAACCGCGGATGA	qRT-PCR
ldtB RT-F	GGTGGCGCGGCGACCCGCCAG	qRT-PCR
ldtB RT-R	GATCTTGATGGCCTTCTCGGC	qRT-PCR
sigA RT-F	GGAGAAGTTCGACTACACCAAG	qRT-PCR
sigA RT-R	GTTGATCACCTCGACCATGT	qRT-PCR

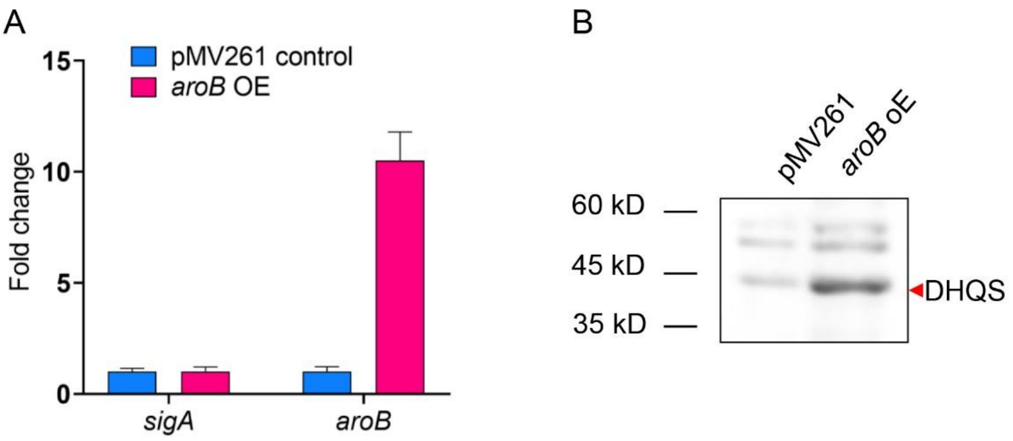

Supplementary Figure 1. The expression of *aroB* in *aroB* OE strain was detected by qRT-PCR (A) and western blotting (B). The pMV261 control and *aroB* OE srains were cultured in streptomycin-free medium for 10 days, then the bacteria were collected for qPCR analysis and western blotting. Error bars represent the standard deviation of three biological replicates.

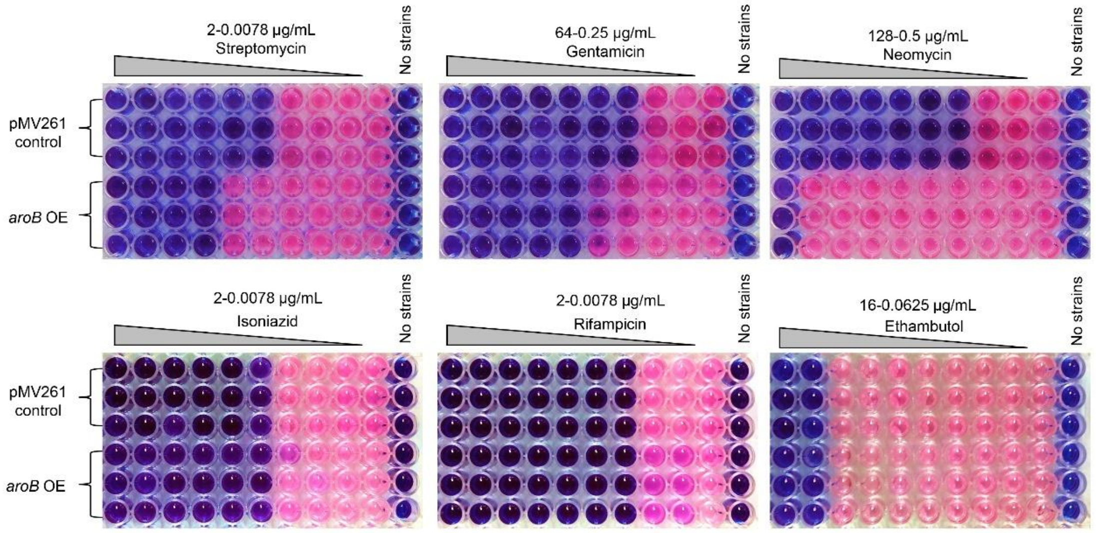

Supplementary Figure 2. The MICs of recombinant *M. bovis* BCG strains against different drugs. The *aroB*-overexpressing and pMV261 control strains were incubated with different concentrations of the several anti-tuberculosis drugs. No strains indicate only 7H9 medium in the Wells. Pink represents bacterial growth. Blue represents bacterial death. The lowest concentration of drug in blue is MIC.

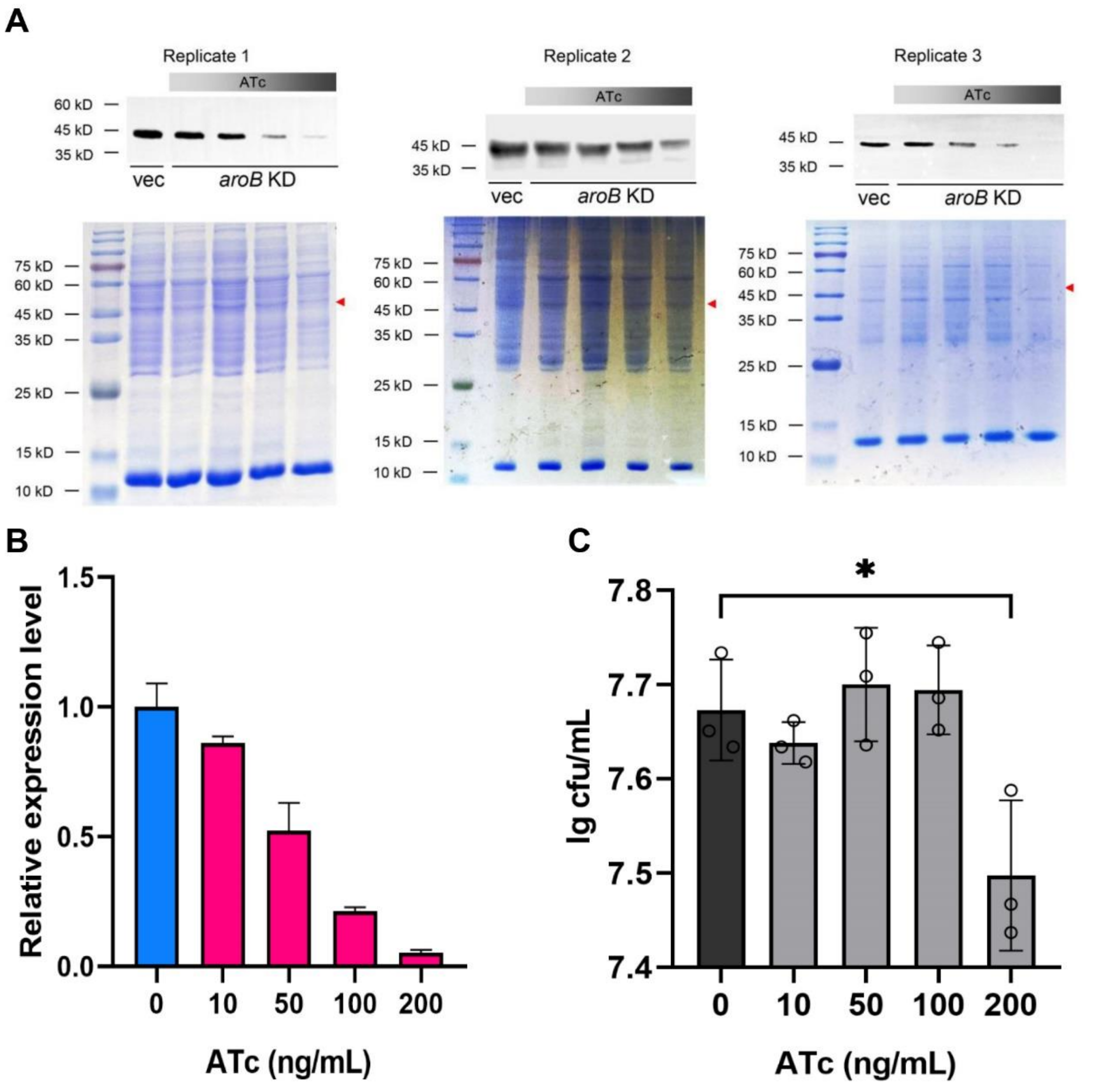

Supplementary Figure 3. Efficiency of CRISPRi-mediated gene silencing was detected by western blot and qRT-PCR. (A) Analysis of DHQS protein expression in *aroB*-knockdown strain by western blotting. *M. bovis* BCG transformed with pLJR965 or aroB sgRNA expression vectors were treated with 10, 50, 100, and 200 ng/mL of ATc. The DHQS protein expression levels were then immunoblotted with anti-DHQS antibody. Coomassie bright blue staining was shown below. (B) Analysis of *aroB* mRNA expression level in *aroB* knock-down strain by qRT-PCR. The expression level of *aroB* in *aroB* knock-down strain was detected under different concentrations (0 ~ 200 ng/mL)

of ATc treatment for 5 days. Signals were normalized to the housekeeping *sigA* transcript for knockdown *M. bovis* BCG gene. The degree of change in relative quantity was calculated by $2^{-\Delta\Delta Ct}$ method. Error bars represent the standard deviation of three biological replicates. (C) CFU count of *aroB* knock-down strain treated with different concentrations ATc for 5 days. Error bars represent the standard deviation of three biological replicates. *P*-values were calculated by GraphPad Prism 8 with an unpaired two-tailed Student's *t*-test. Asterisk represents the significant difference between between the two groups of data ($*P<0.05$).

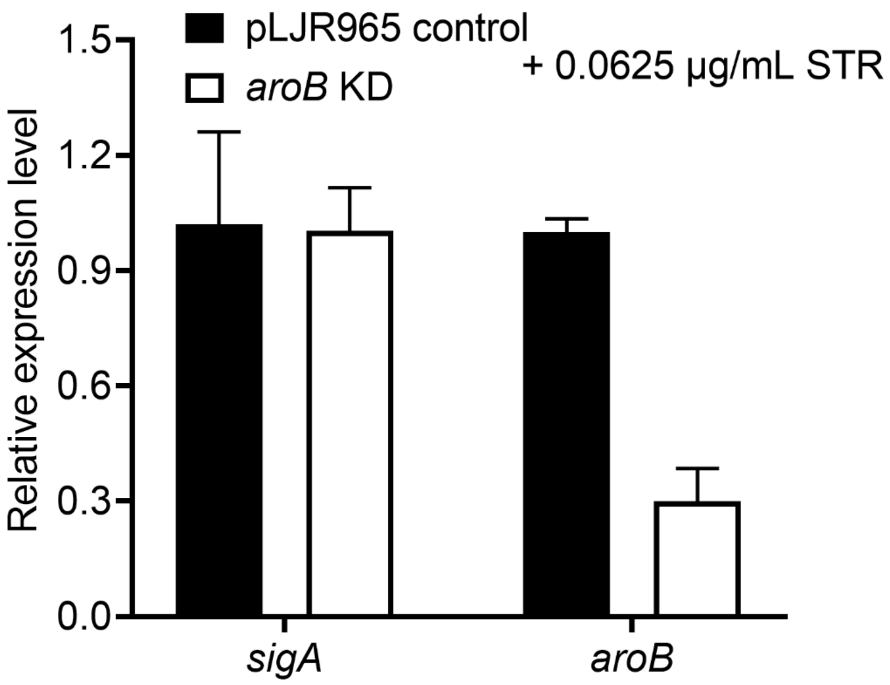

Supplementary Figure 4. Expression level of *aroB* in knock-down strain under streptomycin treatment. The *aroB* knock-down and control strains were cultured in 7H9 medium supplemented with 0.0625 μg/mL streptomycin and 100 ng/mL ATc for 10 days, and then the mRNA level of *aroB* was detected. Error bars represent the standard deviation of three biological replicates.

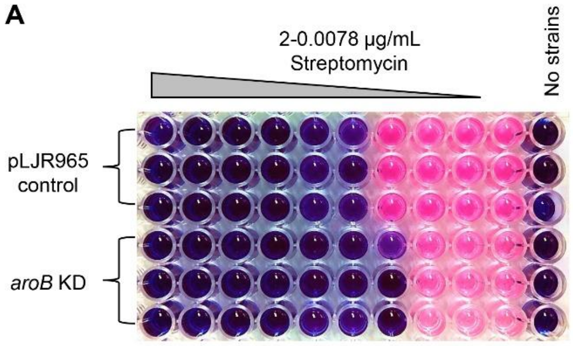

B

Strains	MIC (μg/mL)
	Streptomycin
pLJR965 contraol	0.0625
aroB KD	0.03125

Supplementary Figure 5. The MIC of *aroB* knock-down and pLJR965 control strains against streptomycin. (A) The MIC determination with *aroB* knock-down and pLJR965 control strains. (B) Table of MIC of *aroB* knock-down and pLJR965 control strains against streptomycin. No strains indicate only 7H9 medium in the Wells. Pink represents bacterial growth. Blue represents bacterial death. The lowest concentration of drug in blue is MIC.

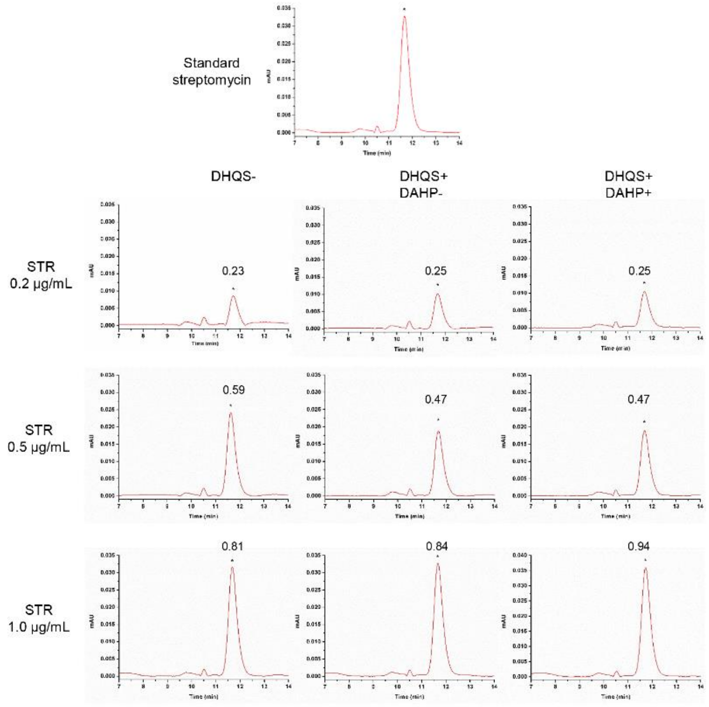

Supplementary Figure 6. DHQS does not modify or hydrolyze streptomycin. The streptomycin at the concentration of 0.2, 0.5, 1.0 μg/mL was incubated with 0.375 μM DHQS or 0.375 μM +140 μM DAHP for 30 min at 37℃. The samples were detected by HPLC and eluted with 12% acetonitrile. The pure streptomycin was used as the positive control (the upper panel) and the characteristic peak for streptomycin is indicated by asterisks. Streptomycin still existed and no new peeks were detected after incubation.

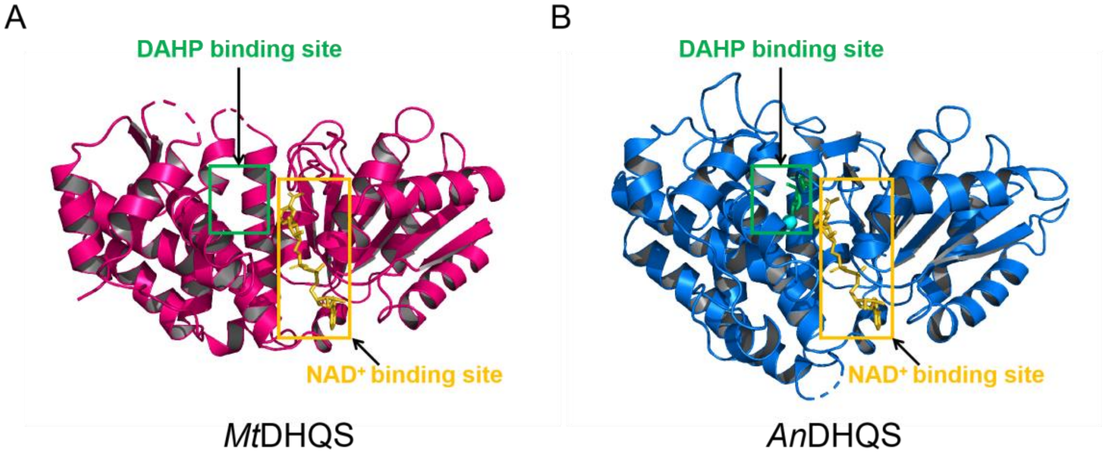

Supplementary Figure 7. Binding sites of NAD^+ or DAHP to DHQS. (A) DHQS of *Mycobacterium tuberculosis*. The NAD^+ binding can be directly identified according to the complexed NAD^+ molecule. The DAHP binding site can be identified by comparing to DHQS structure of *Aspergillus nidulans.* (B) DHQS structure of *A. nidulans.* The structure of AnDHQS contains a DAHP analogue carbaphosphonate in DAHP binding site.

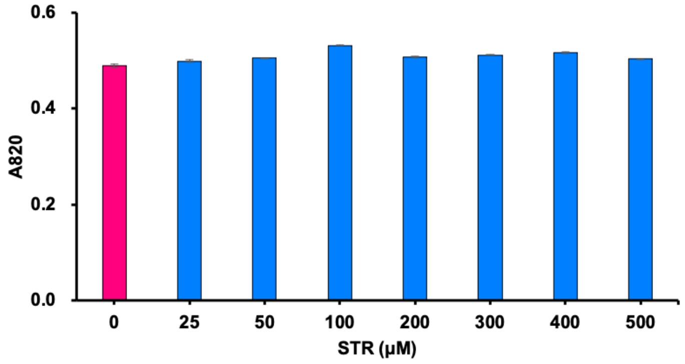

Supplementary Figure 8. Streptomycin has no effect on DHQS enzyme activity. DHQS enzymatic activity is determined by the released inorganic phosphorus content. The catalytic reaction was carried out using 0.4 μM DHQS, 200 μM NAD^+, and 200 μM DAHP in a total volume of 60 μL. The released inorganic phosphorus was combined with ascorbic acid-ammonium molybdate to form a yellow complex and the absorbance at 820 nm were detected. The effect of streptomycin on the enzyme activity of DHQS was determined by adding streptomycin with increasing concentration under the same conditions. The data were expressed as mean ± SDs (error bars) of three biological replicates.

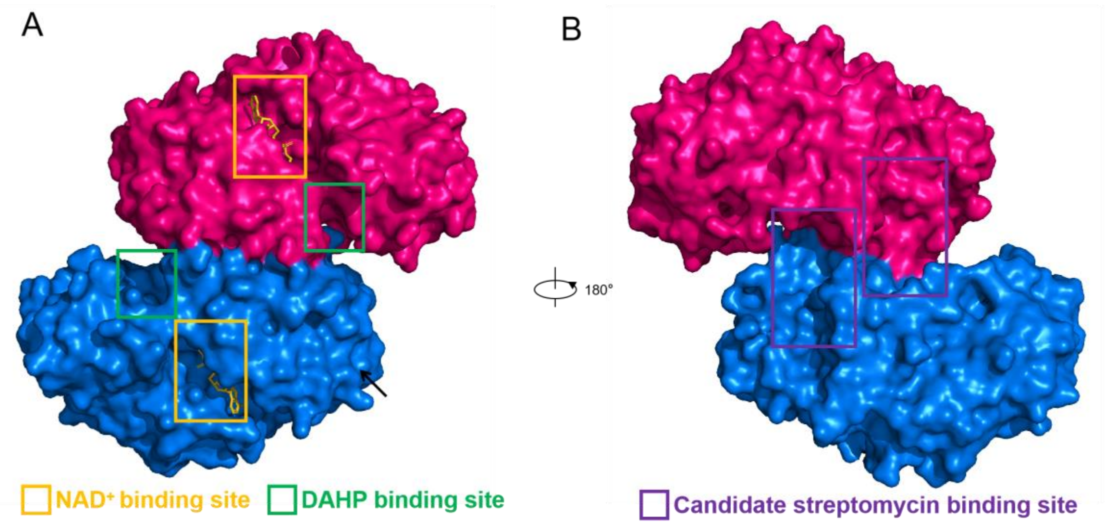

Supplementary Figure 9. Candidate streptomycin binding site. (A) DHQS contains two known ligand binding sites: NAD^+ co-factors and DHAP substrate binding sites. (B) The new identified candidate streptomycin binding site is located on the backside of DHQS comparing to NAD^+ and DAHP binding sites.

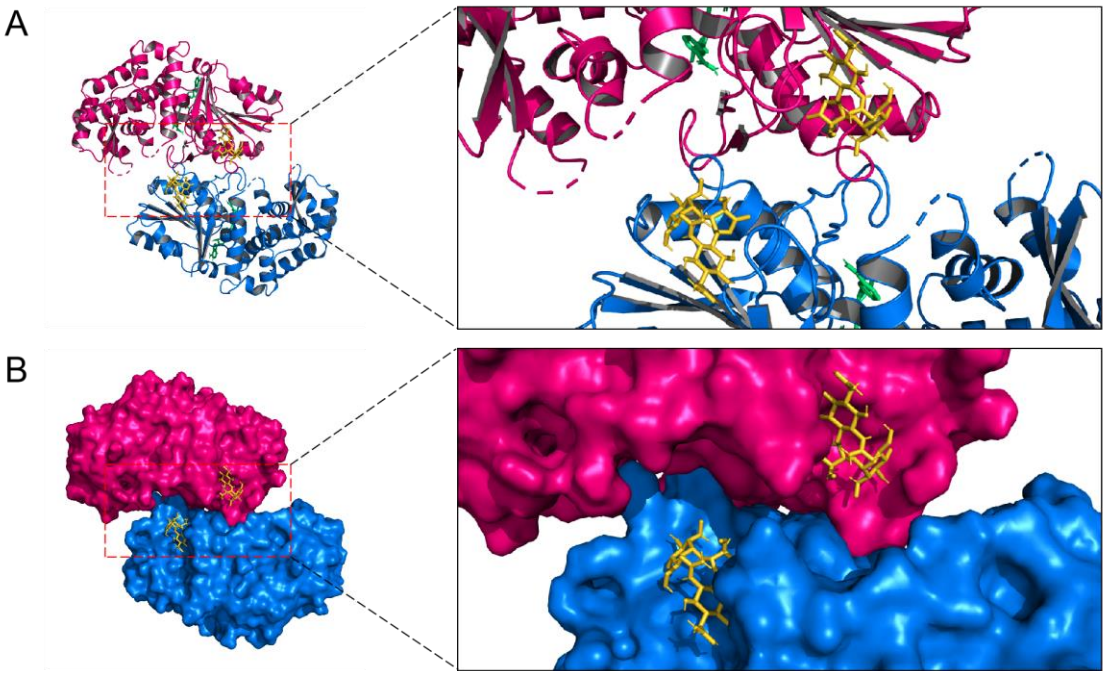

Supplementary Figure 10. Binding model of streptomycin and DHQS. The complex model was modeled by molecular docking. Streptomycin is shown as with sticks representation**.** DHQS binds streptomycin with 1:1 stoichiometry in this model. (A) Dimmer DHQS is shown in cartoon representation. (B) Dimmer DHQS is shown with surface representation.

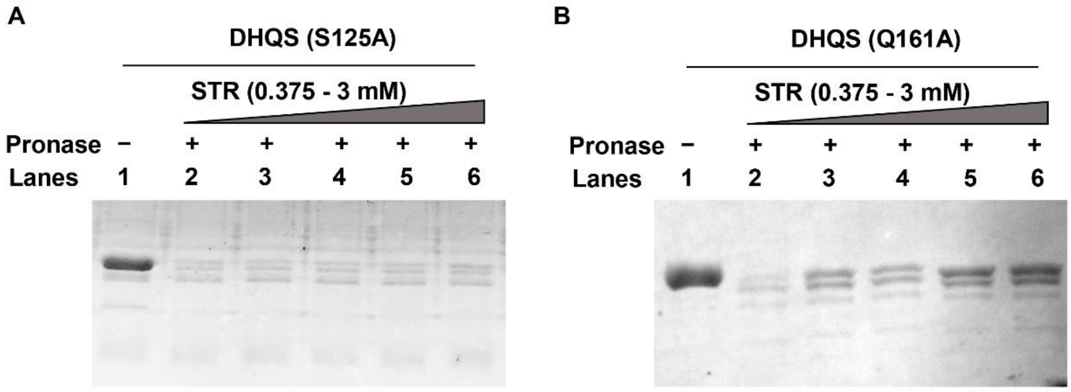

Supplementary Figure 11. Detection of DHQS mutants binding to streptomycin. DARTS detection of the binding ability of DHQS S125A mutant (A) and Q161A (B) to streptomycin.

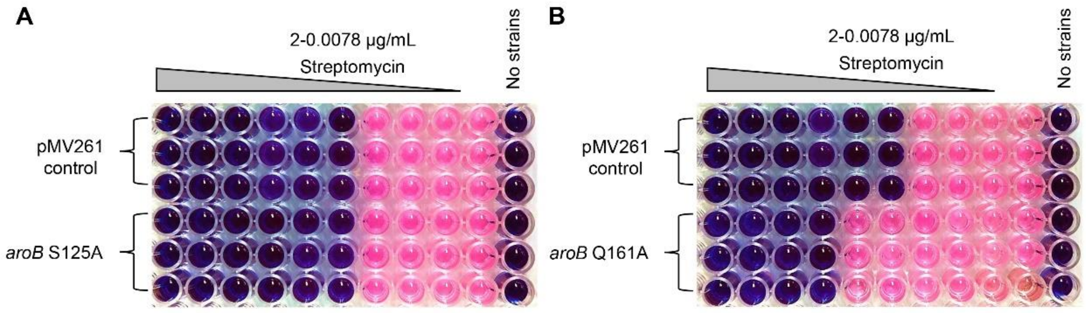

C

Strains	MIC (µg/mL)
	Streptomycin
pMV261 control	0.0625
aroB S125A	0.0625
aroB Q161A	0.25

Supplementary Figure 12. The MICs of *aroB* mutant strains against streptomycin. The MIC determination with S125A mutant strain (A) and Q161A mutant strain (B). (C) The table of MIC of *aroB* mutant and pMV261 control strains against streptomycin. No strains indicate only 7H9 medium in the Wells. Pink represents bacterial growth. Blue represents bacterial death. The lowest concentration of drug in blue is MIC.
